# How Far Can Conversational Agents Contribute to IBD Patient Health Care—A Review of the Literature

**DOI:** 10.3389/fpubh.2022.862432

**Published:** 2022-06-30

**Authors:** Cláudia Pernencar, Inga Saboia, Joana Carmo Dias

**Affiliations:** ^1^ICNOVA—NOVA Institute of Communication, NOVA School of Social Sciences and Humanities, Universidade NOVA de Lisboa, Lisbon, Portugal; ^2^LIDA—Arts and Design Research Lab, Polytechnic Institute of Leiria, Leiria, Portugal; ^3^UFC Virtual, Federal University of Ceará, Fortaleza, Brazil; ^4^DigiMedia—Department of Communication and Art, University of Aveiro, Aveiro, Portugal; ^5^COMEGI—Research Center on Organizations, Markets and Industrial Management, Lisbon, Portugal; ^6^UNIDCOM/IADE—Design and Communication Research Centre, Lisbon, Portugal

**Keywords:** artificial intelligence, chatbot, conversational agents, digital health, IBD, machine learning

## Abstract

Modern societies are facing health and healthcare challenges as never seen before. The digital world in which we are living today considers digital health interventions such as “internet-delivered” therapy (e-Therapy) or mobile apps as an integrated part of healthcare systems. Digital transformation in health care requires the active involvement of patients as the central part of healthcare interventions. In the case of chronic health conditions, such as inflammatory bowel disease (IBD), it is believed that the adoption of new digital tools helps to maintain and extend the health and care of patients, optimizing the course of the treatment of the disease. The study goal was to undertake a literature review associating the use of chatbot technology with IBD patients' health care. This study intends to support digital product developments, mainly chatbot for IBD or other chronic diseases. The work was carried out through two literature review phases. The first one was based on a systematic approach and the second was a scoping review focused only on Frontiers Journals. This review followed a planned protocol for search and selection strategy that was created by a research team discussion. Chatbot technology for chronic disease self-management can have high acceptance and usability levels. The more interaction with a chatbot, the more patients are able to increase their self-care practice, but there is a challenge. The chatbot ontology to personalize the communication still needed to have strong guidelines helping other researchers to define which Electronic Medical Records (EMRs) should be used in the chatbots to improve the user satisfaction, engagement, and dialog quality. The literature review showed us both evidence and success of these tools in other health disorders. Some of them revealed a huge potential for conversational agents as a part of digital health interventions.

## Introduction

Digital health is an umbrella expression that joins health areas allied to digital technology usage. It involves a wide technology landscape and standardized health design for better customized healthcare services. Digital health has an important role in supporting healthcare systems and public health, aiming for more equity in public access, and reaching toward universal health coverage. It uses different delivery systems that connect and interpret data (1, 2).

One of the delivery systems based on computational dialogs is the conversational agents. The conversational agents' era has now become a reality. It is a digital tool that includes software and hardware, “that uses machine learning and artificial intelligence methods to mimic human-like behavior and provide a task-oriented framework involving dialogue” (3). Considering the technologies that are behind conversational agents, otherwise known as chatbots, it is worth noting that machine learning (ML) is the study of computer algorithms that automatically improve the virtual communication experience and is seen as a subset of artificial intelligence (AI), defined as the intelligence demonstrated by the machines.

Looking back to half a century ago, there emerged the programmable natural language ELIZA, developed at the MIT Artificial Intelligence laboratory by Joseh Weizenbaum. It may be considered the first chatbot therapist (4). Years later, the employment of this tool made it possible to build commercial conversational agents, such as Google Home and Amazon Echo. The technology has also been advancing to the point where chatbots incorporate a natural language processing capacity for speech, dispensing with the need for a keyboard, as anyone who uses Siri knows.

In the healthcare environment, these digital tools may offer particular advantages because conversational agents are always available when patients need to interact with them. This programmed machine is never distracted by other issues and always remembers everything (5). This issue enables us to predict patients' needs based on their conversations, purchasing, or browsing behaviors. It is also considered a way of improving value in health care because it promotes the exchange of quality healthcare services and, consequently, better health outcomes for all.

Recent research (3) and media information (6, 7) has shown increasing evidence of conversational agent contributions since 2017, in particular regarding mental health. They are being used in the prevention of suicide and cognitive-behavioral therapy. Considering this type of usage, it should be evident that clinical requirements are well suited to the existing e-Therapy digital tools. This is because a conversational agent involves a dialog system that responds automatically using human language. In addition, e-Therapy provides both internet-delivered data and simple online self-help resources.

Authors such as Gratzer and Goldbloom (5) have classified how e-Therapy offers guidance to physicians on three levels:

- Low—Those patients who are told about websites and/or apps or find them on their own to aid their health condition;
- Medium—Patients are given self-directed tools by their physicians;
- High—Internet-delivered therapy or apps which are integrated into traditional health care systems followed by discussions between patients and physicians.

With reference to the above, it is clear that conversational agents involve high levels of interactions such as programmed dialogs that e-Therapy does not include. It is important therefore to understand the “starting point” of chatbot technology and how it has been introduced into the field of health care.

The way patients are using healthcare mobile apps nowadays is similar to the early days of conversational agents. Within the chatbot technology, Torous et al. (4) have discussed several issues related to chatbots that have been incorporated into mobile apps. First, they have highlighted that there is an increasing number of apps. They said that only one in four revealed a quality standard. Second, when patients download a mobile app, it does not mean that they are necessarily going to use it. Third, downloading a mobile app involves challenges for patients in finding the right app at the right time. Fourth, the majority of users do not understand the ethical issues associated with mobile apps which do not offer the right to privacy (8).

The usage of ML and AI technologies in gastroenterology has been carried out since 2018 (3–11). Only one experimental study was found (12), which was published in May 2020. It was a retrospective cohort study where the authors explored the use of chatbots for patients with IBD. It had also been developed from a poster previously published (12), which was found during the systematic literature review process conducted for this research. These findings demonstrate a huge potential to explore and consider other medical chatbots since there are many different systems where they can be used for multiple purposes, e.g., dementia (13)—the chatbot acts through voice recognition, working as a companion for patients with short-term memory loss, helping physicians to identify signs of the patient's condition; insomnia^1^ – acts through conversations *via* text, working as a companion for insomniacs when they are awake at night; pediatric issues (14)—chatbots are designed to help pediatric patients get appropriate medicine for certain ailments; childhood obesity^2^—acting as a peer companion, using an app interface, for obese teens to keep them engaged through text messages; and psychiatry^3^—to help patients think about their critical situations^4^ (6, 12).

The goal of this study is to systematically review the literature regarding the use of chatbot technology in the healthcare of patient with IBD. This study aimed to support future developments in digital health, mainly chatbot for IBD or other chronic diseases. To this end, several research questions were posed:

- How do academics describe the use of chatbots by healthcare professionals and patients with IBD?
- Who are the researchers that are studying this topic?
- What are the patient profiles that are targeted for using this digital tool?
- What are the implications of using chatbots in the health care of patients with IBD and healthcare professionals?

This document is divided into 5 sections: first, the Introduction, where a summary of the context is given; second, Materials and methods, setting out the search strategy adopted through two different phases and the selection criteria used to filter the articles in both phases: (a) systematic literature review and (b) scoping review; third, Results, where the global outcomes are shown through the (a) PRISMA (see text footnote ^4^) method and (b) description method. In this section, the selected studies are analyzed using the narrative research approach; fourth, the Discussion, in which the impact of using digital tools such as a conversational agent is examined; finally, the Conclusion, with implications for further research.

## Materials and Methods

This literature review study was developed into two phases. First, it was carried out by a systematic approach and the second effort was a scoping review on Frontiers Journals. These are detailed below.

### 1st Phase—Search Strategy and Selection Criteria

First, the team shared their different experiences and perspectives on the subject. This resulted in a title, “How far can conversational agents contribute to IBD patient healthcare—A review of the literature.” Following this, the principal goal was established, “to understand the current implications and possibilities for a chatbot in IBD as a channel of communication for physicians and patients.” This implied the need for a systematic literature review to be carried out. Another point of discussion was a future goal that our team would establish: the development of a pilot scheme for an online platform for IBD management/control that uses ML or AI frameworks, enabling the chatbot to interact virtually with a patient. The purpose of adopting this technology would be, for example, to predict automatically, disease complications, and to support physicians, clinicians, and others.

Following a period of initial discussions, the research team decided to investigate the literature published from 1 January 2014 to 31 December 2019. This task was performed in February 2020 on ACM, PsycINFO, PubMed/NCBI, Scopus, and WOS, using a team of three researchers for the validation process. After that, Parsifal was chosen as the online tool to plan, structure, gather, register, and screen the studies.

The initial brainstorming process of the research team was supported by Parsifal online tool^5^, and it was divided into different phases. First, a plan was carried out that was structured by protocol, objectives of the study, population, intervention, comparison, outcomes, and context (PICOC); research questions; related keywords and synonyms; search string definition; database sources; inclusion and exclusion selection criteria. Second, the research team kept Parsifal and conducted a phase composed of study selection, quality assessment, data extraction, and data analysis.

It is worth mentioning that keywords and synonyms were broadly discussed by the three researchers. Another Parsifal feature that contributed to the aforementioned task was an automatic generation control of a research string. Regarding the brainstorming of keywords (Table 1), synonyms and the PICOC field previously referred to, the “Parsifal” software queried that we included in the databases search the following:

- (“IBD^*^” OR “Crohn's Disease^*^” OR “Inflammatory Bowel Disease^*^” OR “Ulcerative Colitis^*^”) AND (“Chatbot^*^” OR “AI^*^” OR “Artificial Intelligence^*^” OR “Chat^*^” OR “Conversational Agent^*^” OR “Conversational Assistant^*^” OR “Conversational Interfaces^*^” OR “Machine Learning^*^” OR “Messaging Applications^*^” OR “Natural Language Processing^*^” OR “NLP^*^” OR “Question Answering System^*^” OR “Robot^*^” OR “Service Automation^*^” OR “Virtual Agent^*^” OR “Virtual Assistant^*^”).

**Table 1:** Brainstorming of keywords, synonyms, and field.

**Keyword**	**Synonyms**	**Related to (PICOC)**
Chatbot	AI	Outcome
	Artificial Intelligence	
	Chat	
	Conversational Agents	
	Conversational Assistants	
	Conversational Interfaces	
	Machine Learning	
	Messaging Applications	
	Natural Language Processing	
	NLP	
	Question Answering System	
	Robot	
	Service Automation	
	Virtual Agents	
	Virtual Assistants	
IBD Patients	Crohn's Disease	Population
	Inflammatory Bowel Disease	
	Ulcerative Colitis	

The criteria of inclusion and exclusion were defined by discussion between members of the research team. It was decided that studies should be included in the review if they met at least three of the criteria referred to below, and two of them should be “Focused on IBD” and “Focused on the patient.” Criteria for inclusion were as follows:

- Focused on IBD and;
- Focused on the patient and;
- Involved technology related to a chatbot, ML, or AI;
- Involved interventions such as non-pharmacological therapies, multi-component interventions such as complementary strategies to increase adherence to treatments;
- Multiplatform technology (software developed for multiple operating systems);
- Prospective communication studies involving Electronic Medical Records (EMRs).

Studies were excluded if they matched at least two of the criteria referred to below and one of them should be “Study Design.” Duplicated studies, or those which were not written in English, were also excluded, as well as studies whose outcomes did not match the purposes of this investigation. Criteria for exclusion, therefore, were as follows:

- Duplicates or not in English;
- Study design (e.g., the design of clinical trial);
- Clinical experiences;
- Clinical intervention;
- Description of the chatbot for other purposes (conversational agents used without intervention proposes) or future research;
- Focused only on clinical issues (e.g. nutrition, gut microbiota, and immunity).

### 1st Phase—A Strategy Adopted for Data Extraction

As mentioned above, Parsifal helped the research team with both brainstorming and conducting processes until all the data had been collected. After that, it became necessary to register all phases of data extraction (Table 2). Given the complexity of data, the team decided to use Airtable^6^ which is an online tool that is used as a complement to Parsifal. The Airtable sheets were used to globally organize all the research. Each tab corresponds to one research phase and is presented in Table 2. Below, each phase will be explained in detail.

**Table 2:** “Airtable” tabs.

**Label name**	**Screening number**	**Description of the method**	**Not meeting the inclusion criteria**	**Not meeting the exclusion criteria**
01_1stPhase_LRCycles	7,586	Title and abstract screening		
02_2ndPhase_LR_53SelectedArticles	53		X	
03_3rdPhase_LR_30SelectedArticles	30	Full-text screening		X
04_4rdPhase_LR_9ArticlesIncluded	9	Describe the included studies		

The first phase of the data extraction was registered in tab “01_1stPhase_LRCycles.” This phase was conducted to retrieve articles that could inform us about the background and discussion in general. Each study has its title and the abstracts that were double screened by three researchers in a random way. When a decision on inclusion or exclusion could not be made based on the defined criteria, the full text was retrieved. If any question persisted, a fourth person made a final decision. From this primary stage, 53 articles were extracted and manually registered in the tab “02_2ndPhase_LR_53 SelectedArticles” with some guideline notes included.

Second, these 53 articles were filtered by reading again the title and the abstract, and also the introduction and the conclusion. The process also involved two people, with one investigator's opinion being validated by the other. Out of 53 articles, 23 of them were excluded. The process of registration was similar. All titles were manually registered in a new tab, the “03_3rdPhase_LR_ _30SelectedArticles,” including other information in subtabs such as if it was rejected or accepted and what were the criteria of exclusion.

A total of three previously rejected articles were checked again and were included in this review. This is because the research team considered that these articles revealed an interesting and detailed technological approach regarding new IBD strategies for patients' behavioral changes. These three articles are as follows: (a) “Challenges in using real-world clinical practice records for validation of clinical trial data in Inflammatory Bowel Disease: Lessons learned” (15); (b) “Decision-making process in colon disease and Crohn's disease treatment” (16); and (c) “Digital health apps in the clinical care of Inflammatory Bowel Disease: Scoping review” (17).

In short, nine articles were included in this research and were read. Only one researcher manually recorded the findings with reference to the inclusion criteria defined earlier by the team. In the tab, “04_4rdPhase_LR_9ArticlesIncluded”, data relating to title, date of publishing, and the disease or conditions involved with it, were also manually registered.

In the following section, the results will be displayed. First, a global perspective is presented of the outcomes using the PRISMA method, and second, a narrative characterization of the studies is given combining the authors' analyses with the research team's overview. Finally, they will be discussed in Section 4.

### 2nd Phase—Search Strategy and Selection Criteria

With the COVID-19 pandemic declared in March 2020 (18), new challenges in the healthcare systems domain emerged. After researchers concluded the first phase of the literature review, they decided to update this original study and include more recent studies.

Another decision regarding the feasibility of this second phase of the search was to focus only on the Frontiers Journals database. The reason being “Frontiers ranks as the 3rd most-cited publisher among the 20 largest publishers with an average of 4.8 citations per article, an increase from 3.9 citations in the previous year.” (19). Regarding the queried referred before, a few doubts emerge when researchers tried to apply that queried: Should the “IBD” word or “Inflammatory Bowel Disease” expression be used in this second phase of the search? Thus, they decided not to use them as a string setting, but to extend it, reducing the initial query to a unique keyword arrangement, in this case: “conversational agents” or “chatbot.” The reason being the selected range data criteria was “past year.” Following this approach, we would like to focus only on the year 2021 collecting the most recent studies published. After, researchers filtered only articles associated with health topics. The titles were screened through a double review discussion. In case of any questions, they kept an abstract analysis.

### 2nd Phase—Strategy Adopted for Data Extraction

The research team established inclusion criteria through a remote discussion based on discoveries that were found out in the Frontiers website. Unfortunately, any article referring to the IBD topic with a chatbot was not found. Thus, the researchers decided to extend this initial scope and include any article related to other chronic diseases. The title and abstract analysis had the following inclusion criteria:

- Articles related to any chronic disease.

The exclusion criteria were as follows:

- Articles only related to methods assessment.

## Results

### 1st Phase—Systematic Literature Review

**Figure 1:**
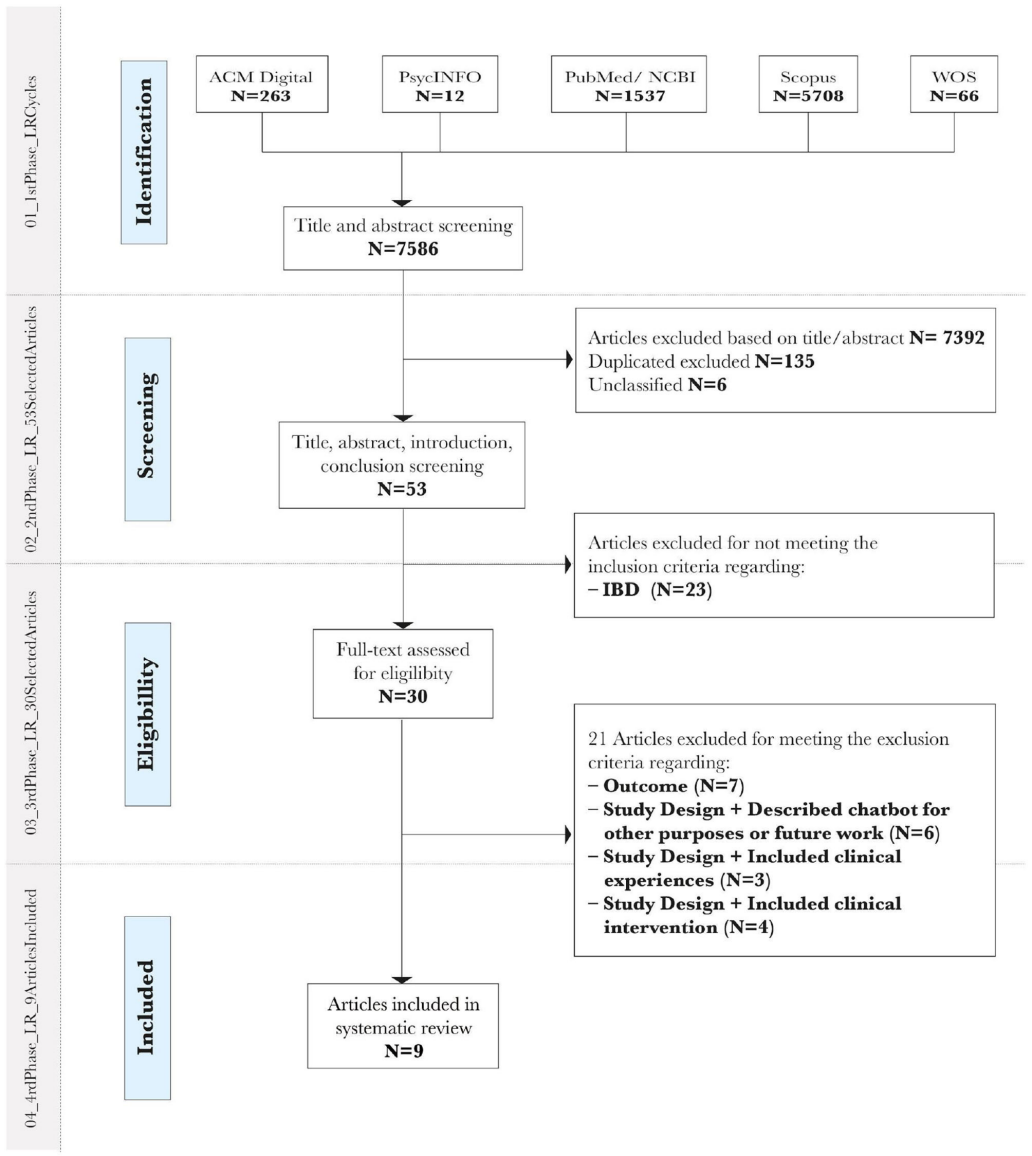
PRISMA flow diagram for systematic review.

The detailed PRISMA diagram in Figure 1 further outlines the number of studies excluded per criteria and matches them with the data presented in Table 2.

In summary, Figure 1 presents the database search retrieved from five databases with a maximum achieved in Scopus *N* = 5,708 and a minimum in PsycoINFO *N* = 12. After that, two researchers' readings went through a more in-depth review process and *N* = 7,392 articles were excluded. From this point, 23 were excluded for not meeting the inclusion criteria “Focused on IBD” and “Focus on the patient,” which is the main context of the review. After a full-text screening of the 30 articles, 21 were rejected for meeting the exclusion criteria detailed in Figure 1. In the end, only nine studies were included in the systematic review. They will be described in detail in part 3.3, Characterization of the studies included.

### 2nd Phase—Scoping Review

This search phase, conducted between 4 January 2022 and 7 January 2022, identified 30 articles without any interval date limitation. The application of two filters resulted as follows: first, 22 articles were published in 2020; second, and after selecting health topic as a domain area, 11 articles emerged as a final result. In the end, this material was spread across these different Frontier Journals categories: digital health (*N* = 7) (20–26); public health (*N* = 2) (27, 28); and psychiatry (*N* = 2) (29, 30). All 11 titles were analyzed through a double review discussion. In case of any issues, the researchers followed the criteria of abstract analysis. There were three articles related to chronic diseases: sickle cell disease (*N* = 1) and mental health (*N* = 2). The last two were related to method evaluation. Therefore, only one article could be added to the first review study.

### Characterization of the Studies Included

In the current section, studies included in the systematic literature review are presented. They come from domains such as computer science, health information, and live science. The presentation of the findings is structured as follows: first, the criteria for inclusion in the study are stated, and second, a narrative review is given. This approach helps the research team to accomplish purposes, such as understanding the results of other studies that are closely related to the one being undertaken and establishing the importance of the findings recognizing where the gaps to be filled may exist.

As a complement to the discussion part, it was decided to create a literature map (Table 3). The goal was to summarize the relevant details of each article not considered in the narrative review.

- Cohn et al. (31)—The criteria to be included in the study were “Focused on IBD,” “Focused on the patient,” and “Involved technology related to chatbot, ML, or AI.”

**Table 3:** Literature map of the included articles.

**References**	**Study type**	**Participation**	**Study duration**	**Intervention and/or methods**	**Outcomes**
Cohn et al. (31)	Experimental study	11 CD patients who underwent MRE between 2010 and 2015	Not applicable to the type of study	CD patients who underwent MRE between 2010 and 2015 and for whom there was at least 6 months of follow-up or an outcome of interest, were retrospectively identified. An expert radiologist demarcated regions of interest (ROI) based on accepted MRE criteria	Radiomics-based ML analysis of MRE (resonance heterography) images of CD patients can be used to develop a personalized risk score to predict response to IS (immunosuppressive) therapy
Afzali et al. (15)	Case studies	867 IBD patients were screened	Not applicable to the type of study	Data from EMRs extracted manually	The screen rate failed in 91.8%
Mossoto et al. (32)	Experimental study	287 children with PIBD	Not applicable to the type of study	Mathematical model assembled different techniques of supervised ML to classify IBD diagnosis in patients with pediatrics	Clinical IBD potential of ML models
Roccetti et al., (33)	Longitudinal study	Crohn's disease experts with a specific pharmaceutical treatment, the infliximab	2 years^a^	Participants post^b^ were read and analyzed by human beings and automatic tools in order to understand their mood when infliximab treatment was mentioned (positives, neutral, or negative term exist in a given discourse)	Gastroenterologists tends to express more positive considerations than the OponionFinder. The non-medical experts tend to return a large number of negatives
Dardzinska and Kasperczuk, (16)	Experimental study	IBD patients	Not applicable to the type of study	The presented model predicts the probability of IBD with malignancy or benign tumors	Classification model tool to find symptoms that affect whether the patients is ill or not^c^
Ashton et al. (34)^26^	Case studies	This study phase is not included in the inclusion of participants	Not applicable to the type of study	Literature review to the analysis of the current management of pediatric IBD applying personalized medicine	AI and ML for personalized medicine^d^
Ashton and Beattie, (35)	Case studies	400 patients in remission until 12 months		Model to predict disease outcome	Identify the potential to translate clinical data from diagnosis into a clinically accurate model predicting the response of medications, complications and others
Borland et al. (36)	Experimental study	This study phase is not included in the inclusion of participants	Not applicable to the type of study		Integrative visualization tool enabling users to explore patients generated research questions or topics
Zand et al. (37)	Experimental study	1712 IBD patients	Do not have this information	Electronic dialog data collected between 2013 and 2018 from a care management platform (eIBD) at a tertiary referral center for IBD at the University of California, Los Angeles (UCLA)	Algorithm showed 94% similarity in categorization compared with our three independent physicians

a *The 261 posts analysis range was between October 2013-October 2015*.

b *Group A – Experts gastroenterologists; Group B – Non-medical experts; OpinionFinder – Standard values into the integer interval (−1,1) based on the algebraic sum of provided scores*.

c *IBD doesn't present a precise diagnosis*.

d *This is the only study founded that refer the potential of personalized medicine within crossing multi-omics data with clinical data (bloods, complications, outcomes, relapse, etc.)*.

This study, presented in poster form, aimed at predicting patients' responses to immunosuppressive therapy by capturing subtle characteristic patterns of the disease in magnetic resonance enterography images. What is interesting in this study, even involving a small sample of participants as few as 11 patients, is the fact that the authors used an ML framework for calculation, by “reading” magnetic resonance enterography images, according to whether the patients were responding, or not, to the immunosuppressive therapy. Their findings showed good feasibility compared with the manual process of analysis conducted by physicians.

In summary, they argued that this method did not represent a risk and could be adopted as a support tool.

- Afzali et al. (15)—The criteria to be included in the study were “Focused on IBD,” “Focused on the patient,” and “Prospective Communication Study involving EMRs.”

The goal of this USA study was to show, through several case studies, what the challenges were regarding the accuracy and the implementation of EMRs. The first case presented in the study pointed to an interesting issue: the source of the data collected is from the Veterans Health Administration (VHA) database; this is restrictive and reveals critical challenges: restrictive, because the type of sample involved did not include women, and challenging because the authors argued that the data collected coming from only one gender. This issue may have produced inconsistent results. Another point was the diagnoses and the treatments of this population were conducted outside the VHA system. This means that there existed more than one database presenting different IBD categories and the systems were not connected between them. Regarding this scenario, some specific data which were not well defined were recorded manually on the VHA database.

The second case reported was PRECISE 3 (38), a study from the Czech Republic. This open-label safety type of clinical trial, in which information is not withheld, aimed to study which were the clinical factors that predicted short-term and long-term efficacy of anti-TNF therapies. PRECISE lasted 7 years and screened 867 patients with IBD. However, the screening process failed in 91.8% of cases because only a small number of participants were eligible. The authors assumed that they believed that these clinical records were initially documented using inadequate clinical variables from heartbeat interval (HBI). In this particular example, it was assumed that there were no differences between the type and the size of the sample considering the percentage of, or reason for, screening failure.

The last case presented was a piece of research conducted by the authors of the article (15), and it was considered an opportunity to reveal more consistent results. It was a retrospective cohort from eight academic and large community practices. It also referred to the challenges with the accuracy of EMRs regarding the high degree of variability in the completeness of EMRs in terms of information resembling data collected during clinical trials.

One of Afzali's team's conclusions was the need for a standardization process to evaluate and document IBD EMRs. Regarding this issue, The American Gastroenterological Association (AGA) has established a document where it documents community practices for IBD regarding the quality and performances to be followed^7^. The authors argued that this orientation was not clear enough on the way IBD EMRs should be carried out.

- Mossoto et al. (32)—This is the first of three selected studies on the subject of personalizing medicine in pediatric inflammatory bowel disease (PIBD) conducted by the same research group. The criteria of inclusion met “Focused on IBD,” “Focused on the patient,” “Involved technology related to chatbot, ML, or AI,” and “Prospective Communication Studies involving EMRs.” This study was published in one of the most prestigious research journals, Nature, in the scientific reports section.

The goal of this study was to present a workflow that aided the accuracy of PIBD diagnostics through a unique experimental approach, being the application of a mathematical model that used different techniques of supervised ML, diagnosing Crohn's disease (CD), or ulcerative colitis (UC) through endoscopic and histological data.

The study involved 287 patients, 178 with CD and 80 with UC, and 29 patients with unclassified diseases from Genetics of Pediatric Inflammatory Bowel Disease at Southampton Children's Hospital in the UK. From this sample, EMRs were collected using a standard platform not identified by the authors. The purpose of this phase was to observe how far clinical features of analysis could induce the development of two data clusters, one for CD and the other, for UC. Concerning classification, the groups identified by each cluster were assessed with reference to “age of onset and C-reactive protein levels at diagnosis, disease subtype, gender, family history and personal history of autoimmune disease (…)” (p. 2). After that, an ML framework was applied to these EMRs aiding initial diagnosis with endoscopy and histology data.

The endoscopic and histological data were collected from 287 patients, but the ML framework was applied only to 239 patients (CD = 143, UC = 67, IBDU = 29) because the model verified that there existed unlabeled data from the validation dataset of 48 participants. The female gender represented 37% (*N* = 107) of the sample in the dataset. The average age was 11.5 years (range from 1.6 to 17.6 years). About 9% (*N* = 26) of patients diagnosed were below 6 years of age (very-early onset of IBD). The remaining 48 patients (CD = 35, UC = 13, the average age of onset being 13.2 years) were used to validate the model.

In summary, the outcome model demonstrates high accuracy in distinguishing CD from patients with UC with a total average of 83.3%: 71.0% through endoscopy data (duodenum, ileum, D-colon, rectum, perianal); 76.9% in histology (ileum); and 82.7% combining both (duodenum, ileum, D-colon, rectum, perianal, esophagus, ileum, and A-colon).

- Roccetti et al. (33)—This article met the inclusion criteria of “Focused on IBD,” “Focused on the patient,” and “Multiplatform technology.”

It is an Italian longitudinal study that aimed to investigate the Facebook posts written by patients with Crohn's disease with particular reference to the effects given by Infliximab treatment.

Over 2 years, 216 posts were analyzed using a social media multiplatform tool. They exhibited opinions written on networking groups with particular reference to the reactions given to Infliximab treatment. The relevance of the findings is that the human side of patients and sentimental issues were investigated in the posts from three different perspectives: group A, composed of experts, e.g. gastroenterologists; group B, by generic assessors without any specific medical competence, and group C with an analysis conducted by a digital tool, OpinionFinder, which is one of the best known and tested ones (34).

The results of the categorization of the posts conducted by the two groups and the software revealed an interesting gap: the expert group tended to assign a negative evaluation to patients' comments on Infliximab with a lower frequency. Only 13% of the posts were assessed as negative by Group A, and 34% of the posts were negatively scored by Group B. Still, the gastroenterologists classified 45% of the posts as neutral in comparison with 42% for the machine result and 25% for non-experts. The values for positive posts were similar between the two groups (Group A−42%; Group B−41%) but, compared with the machine 30%, they were higher.

In conclusion, non-medical experts tend to interpret patients' dialogs with reference to social media posts, more negatively than gastroenterologists. Surprisingly, machines were inclined to agree with the medical experts. This scenario reveals that both types of analyzers, the gastroenterologists and the machine, are only focused on the positive side effects regarding IBD treatments.

- Dardzinska and Kasperczuk (16)—This research met the inclusion criteria of “Focused on IBD,” “Focused on the patient,” and “Involved technology related to chatbot, ML, or AI.”

This Polish experimental study consisted of two phases where a logistic regression method with computerized extraction data was used to classify the CD and UC diseases. One of the two experiments involved ML. The main goal of the study was to present a retrospective analysis of symptoms discovered in medical data that could differentiate UC from CD as quickly as possible in the diagnosis process.

The retrospective analysis of medical data where the authors applied the logistic regression method (a statistical technique to predict values in defined categories) reported interesting results. With a sample of 152 patients with IBD (UC—men *N* = 54 and women *N* = 32; CD—men *N* = 34 and women *N* = 32), researchers used mathematical formulae based on the construction of logic models to classify symptoms that identified if the patients were ill or not. In their investigation, they assumed that the “classification model of the patient is not clear.” Results indicated that even selected attributes from the data did not have a significant impact on the classification of patients' diseases. For these reasons, the authors decided to conduct another phase of analysis using a statistical and ML framework to build a new and improved model, which could more accurately classify a patient's disease. The second case referred to involves a small sample, *N* = 11 patients. Even so, it is a good example of what computerized extraction of data using ML can accomplish. The accuracy of this small mathematical experimental test was 90.9%, whereas a radiologist medical physician could not determine visual differences in the disease that appeared in the image screened because the visualized radiomic features used by radiologists, in almost all digital tools, appear to be marked differently for both scenarios (patients that did and did not respond to treatment).

Briefly, it is hard, using the method explained above, to achieve accuracy regarding disease identification.

- Ashton et al. and Ashton and Beattie (34, 35)—Both studies meet the criteria of inclusion “Focused on IBD,” “Focused on the patient,” and “Involved technology related to chatbot, ML, or AI.”

We decided to include these studies in the same discussion because both cover the same topic. This is an evolutionary process conducted by the same research team regarding the topic of personalized therapy for IBD in children.

The first study (34) reinforced the fact that sophisticated mathematical models and innovative cutting-edge ML techniques give the potential to integrate EMR developing algorithms for personalized clinical care to treat patients more effectively. According to the authors, this process can reduce the “toxicity” existing in the data collected, improving new clinical outcomes and exploring how the future management of IBD may be revolutionized by personalization of clinical care. This study starts by summarizing the current management strategies of treatments used in PIBD.

It helped us to understand how far ML or AI can go if multi-omics data are applied to personalized medicine in IBD. It means introducing biological analysis into the data sets coming from multiple “omics,” such as genomics, proteomics, transcriptomics, epigenomics, metabolomics, and microbiomics.

The aforementioned computational medical challenge regarding personalized IBD therapy is a topic also found in the second study (35). This short paper presents only a reflection on the topic explained before. The authors argue that despite the performance presenting the modeling of single data types, they believed that there is room for improvement by merging diverse data. This means achieving greater power in detecting new IBD subtypes categories using ML.

In summary, with each data type representing a different characteristic of a single patient, ML algorithms can simplify the representation of higher complexity. As a direct consequence, this framework identified a new stratum, which might reflect important clinical outcomes and enable the personalization of therapy based on new groups of multi-omic data.

- Borland et al. (36)—This study met the inclusion criteria of “Focused on IBD,” “Focused on the patient,” and “Multiplatform technology.”

This American experimental study explored how patients think about their health. The goal was to identify, in an online forum and using data visualization, which was the most popular search topic suggested by patients with IBD.

The authors created a website with a discussion forum feature for patients to talk about their IBD experiences. They also created an initial ontology with topics to organize the content in this forum. Crohn's and Colitis Foundation of America (CCFA) was a partner in this project. The foundation was interested in developing efficient approaches to identify new IBD topics of concern for patients and to recognize which search questions were most frequently discussed by them. Regarding the main goal of the study referred to, 97 research topics were identified, and 121 user comments were made by fellow patients on proposed questions, up to a total of 17,322 words.

It is worth mentioning that the initial method of creating the IBD ontology involved a quantitative analysis of the forum data, calculating the frequency of words and phrases. The authors revealed that this method did not effectively capture the nuance of specific lines of research in which the patients were interested. Their solution to overcome this issue was to manually analyze the content by a single person using spreadsheet software and manual data entry.

In total, 165 classes from the Ontology for Adverse Events (OAE)^8^ and 36 from the Disease Ontology (DO) were included. During the ontology creation, IBD partner CCFA was consulted to ensure whether that structure seemed appropriate. The results described a hierarchy of 337 total classes divided by seven top-level groups:

- Comorbidity;
- Diagnosis/monitoring method;
- IBD course—Pre-diagnosis time period, diagnosis event, post-diagnosis time period.
- Quality of life;
- Risk factor—Demographic factor, environmental factor, lifestyle factor, physiological factor, psychological factor.
- Symptom—Gastrointestinal manifestation, extra-gastrointestinal manifestation,
- Treatment method—Alternative therapy, holistic treatment, medication, surgery.

In conclusion, the discussion previously presented helps us to understand which, even in an embryonic phase, are the ontologies created by the authors considering the sample of patients available and the project on its own.

- Zand et al. (37)—This study met the inclusion criteria of “Focused on IBD,” “Focused on the patients,” and “Involved technology related to chatbot, ML, or AI.”

This American study, from UCLA, aimed to explore the use of a conversational agent for IBD health care. It is a short study presented as a poster that demonstrated that the authors were trying to categorize electronic dialog data from patients and healthcare providers through a care management platform including a mobile app.

Regarding the electronic dialogs mentioned, these data were collected from 2013 to 2018. Initially, this information was reviewed manually and after that, the authors created an ML algorithm to categorize the content. The accuracy of this technique was validated by three independent physicians that labeled, manually, 100 lines of randomly picked dialog. Next, they compared the manual process with what the algorithm collected. There was a 94% correlation between the algorithm and the results processed by the three independent physicians. These results show that ML frameworks can achieve similar accuracy, or even higher, compared with a non-automated process of labeling content.

Recently, in May 2020, the same group of authors published a JMIR article (12) where they explained in detail the study stated earlier, highlighting the feasibility of using natural language processing (NLP) for the categorization of IBD EMRs to be used in the development of a chatbot. Although this study is outside the systematic literature review process, some topics will be outlined in the Discussion section because the results inform our research questions in some respects.

- Issom et al. (20)—This study met the inclusion criteria of “articles related to any chronic disease” defined in the second search phase.

The aim of this study was to test the usability and perceived usefulness of the high-fidelity prototype chatbot “TREVOR” which is a part of a mHealth coach app for patients with sickle cell disease (SCD). SCD chronic illness is a genetic blood disorder. It encounters an increasing number of comorbidities. The authors of this article argue that “To our knowledge, no work has been done to design chatbots for the specific self-management needs of people with SCD” (p. 3). This is why they decided to develop “TREVOR” and test the system's usability and usefulness.

The article presents two study phases of robot coach patients' experiences: first, a mixed-methods design research, combining qualitative and quantitative analysis; second, a qualitative survey to understand which was the patients' satisfaction levels and if there were specific recommendations for better conversational agent designing. The sample was composed of 33 SCD participants and 23 were women (medium age is 38 years old). In total, 70% (*N* = 23) of participants were active, whereas 64% (*N* = 21) were affected by the most clinically severe SCD genotypes.

TREVOR was developed using Chatfuel^9^ technology and has been designed to deliver text-based messages and media objects to patients with SCD. The authors of the article explore how this automated health coaching chatbot can improve patients' self-management and support health behavior changes, to understand how to avoid triggering vaso-occlusive crises.

The tests measure the system usability as well as if the robot interaction was empathetic. Its results show us that 73% (*N* = 24) commented positively on how easy it was to use and how fun it was when interacting with the “TREVOR” chatbot. About 82% of patients (*N* = 27) thought the SDC content was useful or interesting. Only 12% (*N* = 4) of patients did not consider the information useful. A total of 18% (*N* = 6) of participants liked how empathetic the chatbot was “It looks like we are communicating with someone who understands our health status” (p. 6). The final survey listed interesting observations: 9.1% (*N* = 3) felt that the content visibility displayed was not optimal; 30% (*N* = 10) requested more flexibility in the choice of answers; 12% (*N* = 4) requested to add more SCD content; 12% (*N* = 4) participants wished to be able to modify their answers more easily.

## Discussion of Principal Findings

### AI or ML With Multi-Omics—A Different Paradigm for IBD EMRs

As seen before, after concluding the systematic literature review, the usage of frameworks such as AI and ML is not new in the IBD environment (32, 34, 35, 37). It has served several purposes but always with the same goal, to help with the classification of the disease, CD or UC.

The other relevant finding in the literature was a growing body of how ML algorithms are being applied to EMRs and how it is changing the accuracy of the data collected (16, 36–38). With reference to this scenario, as seen in the Introduction section, the ML framework can be used in digital tools as a conversational agent. But, to reach this goal, first, it is necessary to analyze the patient profile from different perspectives such as patients' appointments, clinical settings, and others. For us, the literature review was not clear enough about which are the best strategies to adopt since some authors (12, 37) have argued that IBD outcomes can vary. But we found some guidelines that may help us in the future: most of the IBD EMRs differ in certain aspects (12, 15) such as record style, patient behavior, and physician experience from clinic to clinic, as previously explained.

The course of events presented above reflects also that the nature of the data collected is not robust (32). This is because, in the majority of experimental studies reviewed by the research team (15, 32), the IBD EMR collected was categorized manually by experts. Most of these studies did not detail the method used to validate these data and the way experts conducted the process. Only one study (12) mentioned that “doctors evaluate the appropriateness of the categorization by manually categorizing 100 lines of randomly picked dialogue” (p. S244), but we lack more details.

The third finding was the importance of screening the information collected before, identifying which class contents are to be included to define the IBD ontology. Only one study (36) reveals how the authors created an IBD ontology. Furthermore, they argued about the importance of IBD communication being based on different perspectives, patient-to-provider, and patient-to-patient. The authors of the study said, “this is the first such ontology incorporating concepts of using linked views that automatically highlight relationships between selected ontology terms and research topics; the researcher can gain insights into concepts of importance to the forum participants” (p. 384). Apart from this study, IBD ontology is well studied, for example, in the contexts of the nutritional field (39), but no literature was found on IBD ontology for conversational agents. What we discovered was architecture information developed as an initial proposal for a prototype related to semantic technology for IBD. This initial proposal was developed by a group of Chinese researchers (40), but the study presented was at an early stage.

Finally, creating the semantic categories will be important to support intelligence features for the verbal interaction between a conversational agent and patients. With reference to the process of creating semantic categories, the literature (12, 34, 35) indicated that this involves two types of ML: the supervised ML which means that the algorithm predicts the class to which data elements belong, or the unsupervised ML, which uses data which is not classified, categorized or labeled, allowing a more complex analysis than using supervised ML. Even so, we did not discover a detailed list of semantic categories regarding European patients with IBD.

### Conversational Agent—Is it a new Paradigm for Chronic Disease Patients' Care?

As seen, AI and ML frameworks open new opportunities in IBD by improving the accuracy of EMRs manually managed after being collected. An example of this is what Z and his research team (12, 37) are trying to do. Their study from 2020 collected and analyzed 16,453 lines of dialog extracted from the UCLA IBD database and processed manually on a common sheet. After that, 8,324 messages from 424 patients were studied. The first 400 lines of each were manually reviewed defining seven categories by their frequencies:

- Medications (38.70%);
- Communications (34.89%);
- Laboratory investigations (34.01%);
- Symptoms (32.83%),
- Appointments (24.51%);
- Miscellaneous (10.08%);
- Procedures (9.96%)
- Finance or insurance (7.22%).

The keywords used in the algorithm come from these 400 lines—a simplified bag-of-words model. Roughly 90.00% of dialogs that came from patients fell into only seven categories, which shows potential for developing a chatbot with a Neuro-Linguistic Programming (NLP) algorithm that can handle the most relevant IBD patients' questions and concerns. This study presented an interesting flowchart explaining the inclusion and the categorization of dialog, but it revealed something critical that helps us to comprehend why it is so hard to create patterns for IBD ontology: their patient sample was fairly homogeneous, consisting mostly of young (mean age 42 years) and white patients, which limits the extrapolation of our results to other populations.

Sickle cell disease (20) article reveals contributions to IBD study. Chatbot technology for chronic disease self-management can have high acceptance rates and usability scores. The more patients interact with a chatbot, the more knowledge and information the chatbot can support, increasing the self-care practices in an empathetic way.

Principal findings show us that delivery systems, such as chatbots, could be created to have an empathetic personality, and their communication could be more personified as a digital health strategy to improve user satisfaction, engagement, and dialog quality.

## Conclusion and Implications for Research

Regarding the research questions that guided the systematic literature review presented and after concluding the process, our team concludes that the academics are not yet discussing vigorously the use of chatbots by health professionals and patients with IBD, as seems to be happening in other clinical fields such as dementia (13) and pediatric issues such as obesity (14). Considering the IBD topic, only one study was found (12). The authors from UCLA Center For Inflammatory Bowel Disease published an exploratory study in May 2020 about the feasibility of using NLP for the categorization of electronic dialogs. This study reveals how hard it is to define linguistic patterns because the collected data from several dialog sources may influence how these patterns are defined.

We conclude that it will be a challenge to create the IBD ontology because there are no strong guidelines to help researchers to define which IBD EMRs should be used effectively to define linguistic patterns. We have discovered that biological data should be included and also that experts such as gastroenterologists must be included in the data validation process.

Conversational agents in IBD patient care show promise: e.g., in automating requests regarding booking and cancellations, or even by playing an instrumental part in disease triage; following the same guidelines as nurses; and saving the provider team valuable time that could be redistributed to better patient care. However, more experimental studies are needed to achieve meta-analysis and to determine the effectiveness of this digital tool in IBD.

We challenge the research communities to focus their studies on identifying the class content and the linguistic patterns to be included in conversational agents in the IBD ontology, and on creating the semantic categories supporting intelligence features for verbal interaction between a conversational agent and patients with IBD in the digital world.

## Data Availability Statement

The original contributions presented in the study are included in the article material, further inquiries can be directed to the corresponding author.

## Author Contributions

CP and IS contributed to the planning, conducting of the study, and approved the final version to be published. CP, IS, and JD was carried out the search procedure and reporting of the manuscript. All authors contributed the analytic strategy to achieve the final classification of assessment criteria and critically wrote and revised the manuscript providing insights into the review discussion. All authors contributed to the article and approved the submitted version.

## Funding

This work was funded by the National Funds through FCT —Fundação para a Ciência e a Tecnologia under project Ref^a^: UIDB/05021/2020 and Ref^a^: UIDB/04005/2020.

## Conflict of Interest

The authors declare that the research was conducted in the absence of any commercial or financial relationships that could be construed as a potential conflict of interest.

## Publisher's Note

All claims expressed in this article are solely those of the authors and do not necessarily represent those of their affiliated organizations, or those of the publisher, the editors and the reviewers. Any product that may be evaluated in this article, or claim that may be made by its manufacturer, is not guaranteed or endorsed by the publisher.

## Footnotes

^1^https://insomnobot3000.com/ (Accessed January 04, 2022).

^2^https://mobilecoach.com/ (Accessed January 04, 2022).

^3^https://woebot.io/ (Accessed January 04, 2022).

^4^http://www.prisma-statement.org/ (Accessed January 04, 2022).

^5^https://parsif.al/ (Accessed April 30, 2022).

^6^https://airtable.com/ (accessed April 30, 2022).

^7^https://gastro.org/practice-guidance/quality-and-performance-measures/ (accessed January 07, 2022).

^8^http://www.oae-ontology.org/ (accessed January 04, 2022).

^9^https://chatfuel.com/ (accessed December 20, 2021). Chatfuel is the leading chatbot development platform for Facebook Messenger that allows to design fully automated and script-based conversational agents.
